# An Ensemble Based Top Performing Approach for NCI-DREAM Drug Sensitivity Prediction Challenge

**DOI:** 10.1371/journal.pone.0101183

**Published:** 2014-06-30

**Authors:** Qian Wan, Ranadip Pal

**Affiliations:** Electrical and Computer Engineering, Texas Tech University, Lubbock, Texas, United States of America; University of California, Irvine, United States of America

## Abstract

We consider the problem of predicting sensitivity of cancer cell lines to new drugs based on supervised learning on genomic profiles. The genetic and epigenetic characterization of a cell line provides observations on various aspects of regulation including DNA copy number variations, gene expression, DNA methylation and protein abundance. To extract relevant information from the various data types, we applied a random forest based approach to generate sensitivity predictions from each type of data and combined the predictions in a linear regression model to generate the final drug sensitivity prediction. Our approach when applied to the NCI-DREAM drug sensitivity prediction challenge was a top performer among 47 teams and produced high accuracy predictions. Our results show that the incorporation of multiple genomic characterizations lowered the mean and variance of the estimated bootstrap prediction error. We also applied our approach to the Cancer Cell Line Encyclopedia database for sensitivity prediction and the ability to extract the top targets of an anti-cancer drug. The results illustrate the effectiveness of our approach in predicting drug sensitivity from heterogeneous genomic datasets.

## Introduction

The ability to accurately predict sensitivity to anti-cancer drugs based on genetic characterization can assist us in selecting drugs with high chances of success for cancer patients. A number of approaches have been proposed for drug sensitivity prediction. For instance, statistical tests have been used to show that genetic mutations can be predictive of the drug sensitivity in non-small cell lung cancers [1]. In [2], gene expression profiles are used to predict the binarized efficacy of a drug over a cell line with the accuracy of the designed classifiers ranging from 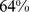 to 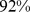. Tumor sensitivity prediction has also been considered as (a) a drug-induced topology alteration [3] using phosphor-proteomic signals and prior biological knowledge of generic pathway and (b) a molecular tumor profile based prediction [1], [4]. Supervised machine learning approaches using genomic signatures achieved a specificity and sensitivity of higher than 70% for prediction of drug response in [5]. In [6], a Random Forest based ensemble approach on gene expression data was used for prediction of drug sensitivity and achieved an 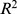 value of 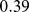 between the predicted 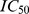
s and experimental 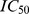
s for NCI-60 cell lines.

However, the methodology for converting the genetic measurements to predictive models for assisting therapeutic decisions still remains a challenge [7]. Detailed dynamical models of genetic regulatory networks [8], [9] are not well suited to predict the tumor sensitivity to kinase inhibitors as the data requirements for model parameter estimation are significantly higher in terms of number of samples and preference for time series data [10], [11]. In the recent cancer cell line encyclopedia (CCLE) study [7], the authors characterize a large set of cell lines (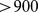) with numerous associated data measurement sets: gene and protein expression profiles, mutation profiles, methylation data along with the response of around 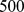 of these cells lines across 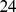 anti-cancer drugs. For generating predictive models, the authors considered regression based analysis with elastic net regularization across input features of gene and protein expression profiles, mutation profiles and methylation data. The performance (as measured by Pearson correlation coefficient between predicted and observed sensitivity values) of the predictive models using 10 fold cross validation ranged between 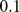 to 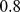. We have recently reported that the prediction can be significantly improved if the drug target profile information is incorporated in the predictive model [12].

In this article, we consider a drug sensitivity prediction approach from heterogeneous genomic datasets that was applied to NCI-DREAM Drug Sensitivity prediction sub-challenge 1 [13] with high performance. For the NCI-DREAM Drug Sensitivity prediction sub-challenge 1, genomic characterizations were provided for 53 cell lines and responses to 31 drugs were provided for 35 of these 53 cell lines. The challenge consisted of predicting the rank order from the most sensitive to the least sensitive of the remaining 18 cell lines for each drug. Our framework consists of generating random forest based ensemble prediction from each genomic dataset (such as RNAseq, methylation, protein abundance) and combine them using a linear regression approach to generate the integrated prediction results. The prediction accuracy as measured by bootstrap error for different combination datasets shows the efficacy of our framework in increasing the prediction accuracy by using multiple datasets. We also applied our framework to the CCLE database and achieved higher prediction accuracy as compared to the elastic net based approach considered in [7].

The article is organized as follows: the *Results* section presents the performance of our framework in the NCI-DREAM drug sensitivity sub-challenge 1 along with subsequent detailed analysis of the challenge provided datasets and CCLE datasets; the *Discussion* section provides the inferences from the analysis along with future directions and the detailed framework is explained in the *Material and Methods* section.

## Results

### The NCI-DREAM Drug Sensitivity Prediction Sub-Challenge 1

In this anti-cancer drug sensitivity prediction challenge, a total of 53 cell lines (48 breast cancer cell lines and 5 non-malignant breast cell lines) were exposed to 31 therapeutic compounds at a concentration required to inhibit proliferation by 50% after 72 hours (GI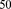) [14]. Multiple types of genomic and epigenetic data (copy number variation, methylation, gene expression through microarray, RNA sequencing, exome sequencing and protein abundance) were generated before exposure of the cells to the drugs for each of the 53 cell lines. The challenge participants were provided with the genomic characterization of 53 cell lines, the 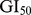 concentrations for 31 compounds (the identity of the drugs were kept anonymous) on 35 cell lines and a list of the 18 cell lines whose corresponding GI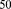 concentrations were not supplied. Meanwhile, within the training data, all the drug responses or genomic characterization could not be reliably measured due to technical reasons. The data was provided pre-publication by Prof. Joe Gray from Oregon Health & Science University.

The challenge consisted of generating a model capable of ranking the sensitivity of 18 breast cancer cell lines and placing them in the proper order to produce a final ranked list of 53 cell lines for each of the 31 compounds. Since the 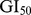 concentrations in the training data are - 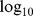 transformed, the lowest ranking (1,2,3…) corresponds to the highest 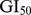 values and the highest ranks (…,51,52,53) correspond to the lowest 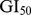 values. The challenge organizers had recommended to place the cell lines with *NA* values at the end of the list and sort them arbitrarily. The description of the NCI-DREAM drug sensitivity sub-challenge 1 genomic and drug response datasets are shown in tables 1 and 2 respectively. From table 1, we note that the genomic characterizations were not available for all the 53 cell lines and each dataset had missing information for some of the cell lines (the number of such cell lines is denoted by *Missing cell lines* in table 1). The last column denotes whether the genomic dataset had some missing values for the cell lines containing that specific genomic characterization.

**Table 1:** Description of Genomic Datasets for NCI-DREAM drug sensitivity challenge. Out of 53 cell lines, 35 cell lines are used for training and 18 for testing the prediction accuracy.

Data Type	Dimension	Missing cell lines	Missing values (Y/N)
Gene Expression	46×18632	7	N
Methylation	41×27551	12	N
RNA seq	44×36953	9	Y
SNP6	47×27234	6	Y
RPPA	42×131	11	N

**Table 2:** Description of Drug Response Data for NCI-DREAM drug sensitivity challenge.

Type of Data	GI50 [14]
Number of Compounds	31
Number of Cell Lines for Training	35
Number of Cell Lines for Testing	18

### Integrated Ensemble based approach to prediction for NCI-DREAM Sub-Challenge 1

Our sensitivity prediction approach consists of a weight-based integrated Random Forest (RF) model to appropriately utilize the information in different datasets. Since the genomic characterizations consisted of numerous features, an ensemble approach such as RF that can utilize the top features based on bootstrap aggregation is expected to have good performance. A regularization approach on linear regression such as elastic net can limit the number of features but may lack in accuracy due to the nonlinear interactions among genomic features.

In our modeling approach, we initially generated single RF regression models based on each data type and subsequently applied least square regression to estimate the proportional weight of each individual model. Additionally, for initial validation of DREAM-Challenge prediction results, we used leave-one-out (LOO) error estimation to calculate prediction errors of each individual RF model and the model with the integrated RFs. Even though LOO error estimation may have high variance from the true error, we selected LOO for the DREAM challenge submission due to time complexity considerations (as compared to bootstrap error estimation) and the availability of small number of samples where holding out more samples may degrade the model estimation significantly. However, we have applied bootstrap error estimation later on for our detailed analysis of the datasets. For the challenge submission, we selected the model (from among the individual and combined models) with minimum prediction error (based on leave-one-out error estimation) as our final prediction model for each drug. Instead of ranking *NA* values arbitrarily as recommended in the challenge, we treat them as new unknown drug sensitivities to be predicted and rank them according to our prediction.

**Figure 1:**
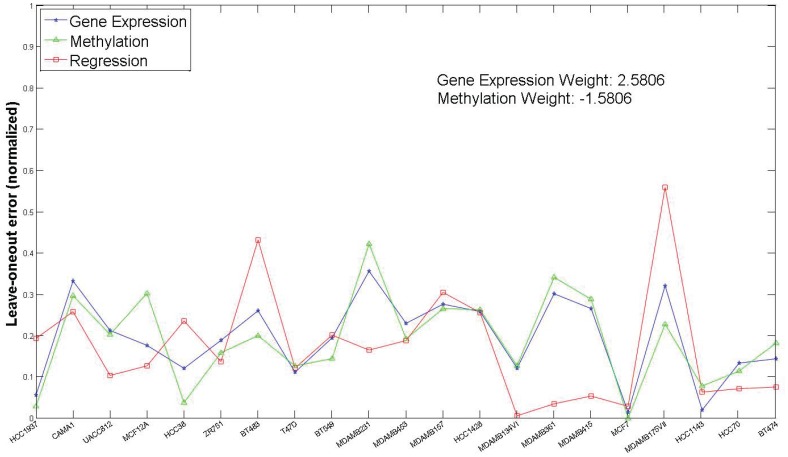
Predictions based on each data type (Gene Expression and Methylation) compared to joint prediction (Regression) for Drug 10.

**Figure 2:**
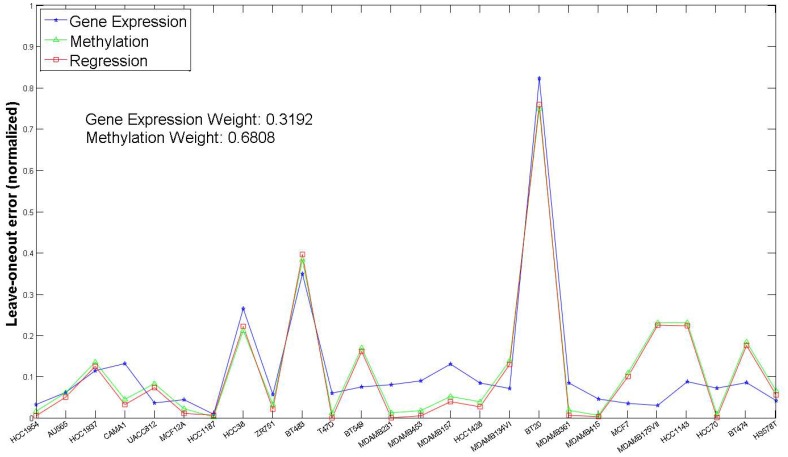
Predictions based on each data type (Gene Expression and Methylation) compared to joint prediction (Regression) for Drug 21.

**Figure 3:**
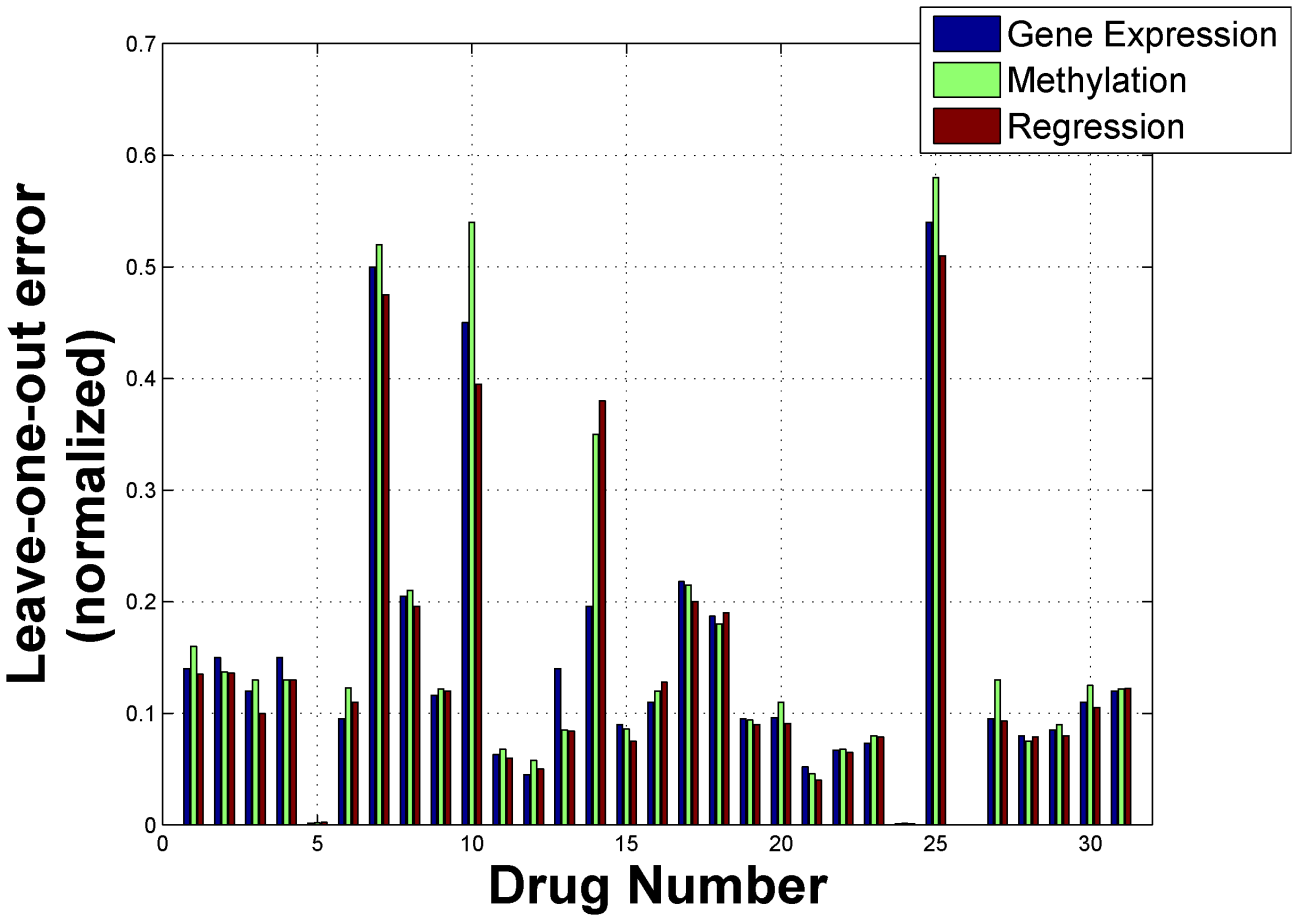
Prediction accuracy comparison for different models for DREAM challenge submission. Two individual models based on gene expression and methylation is considered along with the joint model (Regression) of the two genomic characterizations.

As mentioned previously, we generated predictions from multiple genomic characterizations using RF and then combined them through a linear regression model to extract the predictive information from each dataset. Figures 1 and 2 shows the comparison in terms of LOO prediction errors for combined RF model and individual RF models for two drugs 10 and 21 respectively. For the NCI-DREAM challenge submission, we used the gene expression and methylation dataset for individual models (termed *Gene Expression* and *Methylation* in figures 1 and 2) and combined them through a linear regression approach (termed *Regression* in figures 1 and 2). The analysis incorporating the other datasets is included in a later section. The reference implementation of Random Forests in MATLAB (Windows Pre-compiled version [15]) was used for the individual models. The drugs 10 and 21 (whose detailed performance is shown in figures 1 and 2) were selected randomly. The X-axis in figures 1 and 2 represents the cell lines that had both gene expression and methylation data for the respective drug. Figure 1 illustrates that reduction in error (as estimated by LOO error estimation) can be achieved by applying the combined regression RF model (e.g. cell lines *UACC812, MCF12A, MDAMB361* and *MDAMB134VI*). Figure 3 shows the normalized leave-one-out errors for prediction using gene expression data (blue bars), methylation data (green bars) and the combined gene expression and methylation data (red bars) for all the 31 drugs. Although, the majority of the combined predictions have lower leave-one-out errors than the corresponding individual random forest predictions, a lower leave-one-out error can be achieved through individual prediction for few of the drugs (e.g. Drug 14, Drug 18). The higher estimated error for the joint model may be caused by the high variance of the leave-one-out error estimation. For final prediction of the holdout testing datasets, we selected the prediction model (gene expression, methylation or combined regression) with the minimum leave-one-out error.

**Figure 4:**
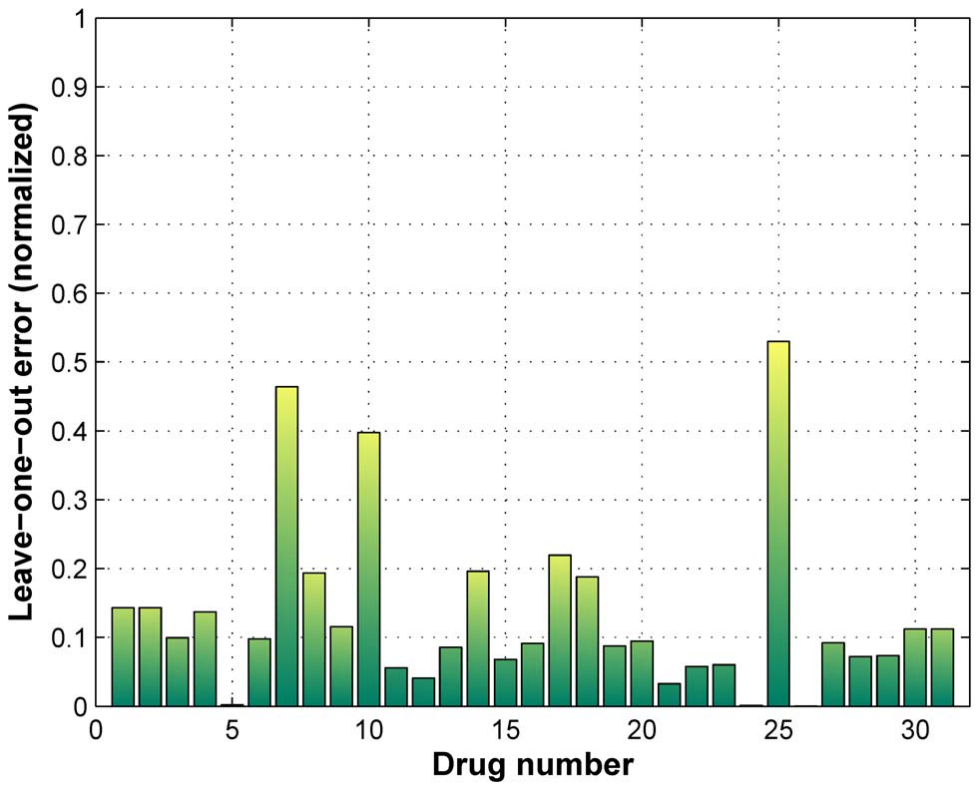
Leave-one-out errors for each drug with integrated random forest model using gene-expression and methylation datasets.

Figure 4 shows the average leave-one-out normalized errors corresponding to the 31 drugs. As figure 4 shows, the majority of errors are around or below 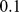 or 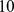 % which possibly denotes that the generated regression model has high accuracy and is appropriate for prediction of new drugs. As the drug response GI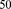 values provided for drugs 5, 24 and 26 are constant, their prediction errors approach zero. Our final drug sensitivity rankings along with the gold standard rankings are included as Information S1.

### Comparison with other approaches and NCI-DREAM drug sensitivity prediction challenge results

We next present the performance of our approach as compared to other submitted approaches. 47 different teams submitted their predictions for this challenge and the performance of the top 10 teams are reported in Table 3. The teams were ranked according to their weighted average probabilistic c-index score (wac-index) [13] which is described in the methods section. The framework applied by *teamfin* and our team (*Holmes*) achieved the best prediction accuracy and were relatively ranked first and second. The rankings of *teamfin* and *Holmes* were statistically found to be significantly robust to perturbations of the gold standard (the p-value and FDR for the first two teams are distinctly smaller than the others). There was a second round of evaluation on 12 more compounds where our approach performed better than *teamfin* in terms of wac-index and p-value (results not included).

**Table 3:** Prediction accuracy and statistical significance of results provided by NCI-DREAM Organizers [13].

Team	Rank	wac-index	p-value	FDR
teamfin	1	0.583	5.47E-07	2.57E-05
Holmes	2	0.577	3.05E-06	7.17E-05
Team#352	3	0.570	2.06E-05	2.91E-04
Team#382	4	0.569	2.48E-05	2.91E-04
Team#383	5	0.565	6.88E-05	5.11E-04
Team#421	6	0.564	7.83E-05	5.11E-04
Team#354	7	0.564	7.92E-05	5.11E-04
Team#384	8	0.564	9.14E-05	5.11E-04
Team#571	9	0.564	9.78E-05	5.11E-04
Team#480	10	0.562	1.24E-04	5.46E-04

### Additional Analysis of DREAM-Challenge Datasets

**Figure 5:**
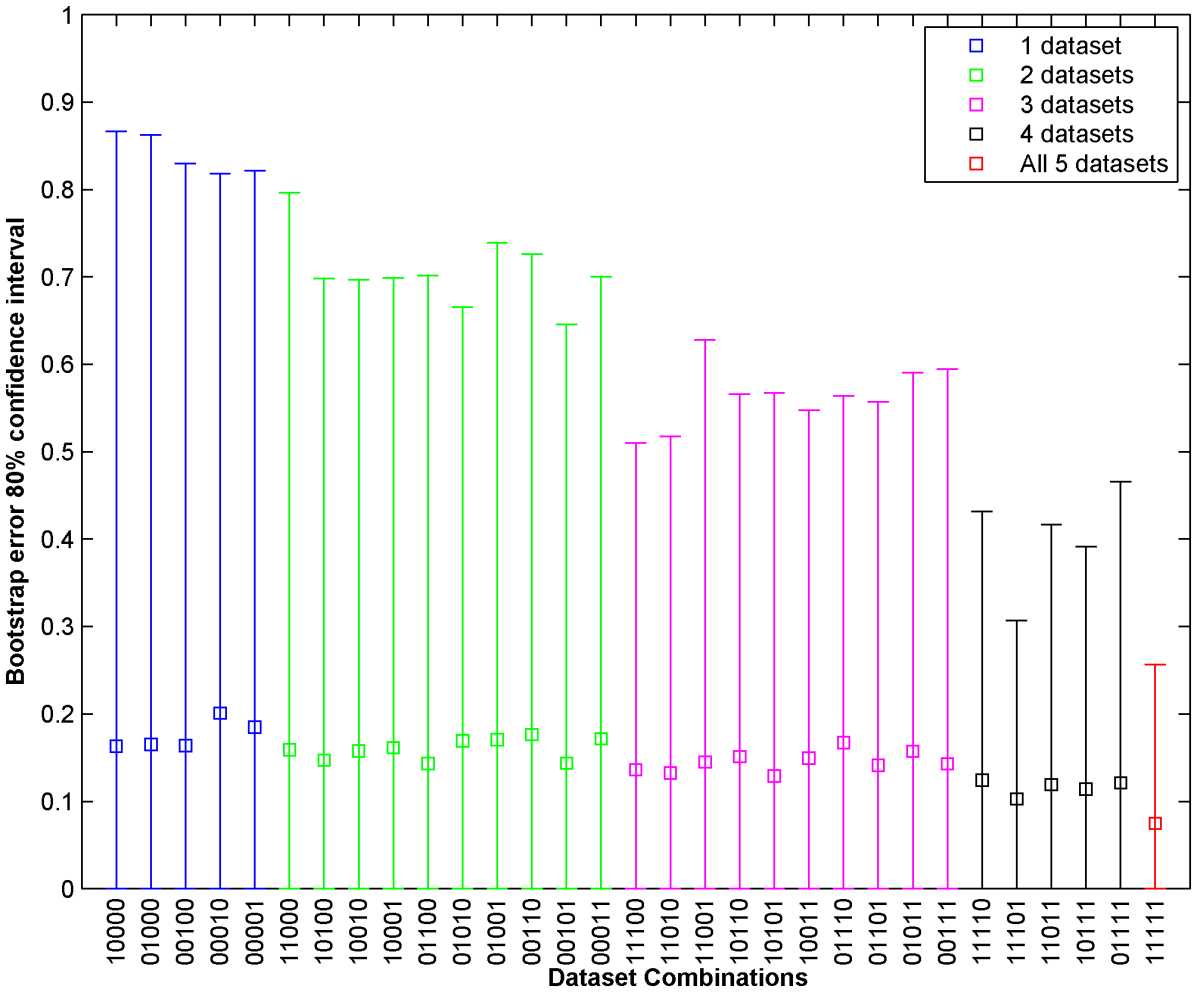
Mean 0.632 Bootstrap error and 80% confidence intervals for Drug 1 for 31 ( = 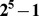) different dataset combinations. The datasets are denoted by binary digits with the following order: Gene Expression (most significant bit), Methylation, RNASeq, SNP6 and RPPA (least significant bit). For instance, 01100 denote Methylation and RNASeq data combination.

**Figure 6:**
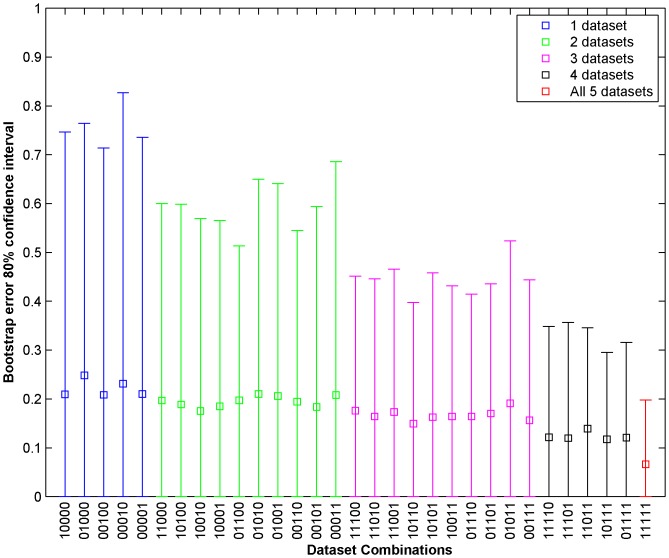
Mean 0.632 Bootstrap error and 80% confidence intervals for Drug 10 for 31 ( = 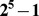) different dataset combinations. The datasets are denoted by binary digits with the following order: Gene Expression (most significant bit), Methylation, RNASeq, SNP6 and RPPA (least significant bit). For instance, 01100 denote Methylation and RNASeq data combination.

In this sub-section, we present results using 5 datasets for prediction and utilizing 0.632 bootstrap for estimating the prediction error. We consider the following five types of genomic characterizations: Gene Expression, Methylation, RNASeq, SNP6 and RPPA (as shown in Table 1). For genetic mutation information, we used the SNP6 data and didn’t consider the additional Exome sequence data for prediction analysis. The confidence intervals for our estimated errors are generated using Jackknife-After-Bootstrap approach [16], [17]. Since we considered mean absolute error (MAE), the lower limit of the confidence interval is kept at 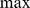 (0, MAE 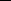 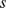
*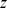) where 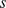 is the standard error estimated using Jackknife-After-Bootstrap approach (see *Methods*) and 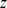 is the specific quantile of the standard normal distribution. Based on 5 datasets, there are possible 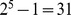 non-null combinations of the datasets. Figure 5 shows the mean 0.632 Bootstrap error and 80% confidence intervals for Drug 1 for the 31 different dataset combinations. The datasets are denoted by binary digits with the following order: Gene Expression (most significant bit), Methylation, RNASeq, SNP6 and RPPA (least significant bit). For instance, 10010 denote Gene Expression and SNP6 data combination. Similarly, figure 6 shows the mean 0.632 Bootstrap error and 80% confidence intervals for Drug 10 for the 31 different dataset combinations. The figures 5 and 6 shows that the prediction error as estimated by 0.632 Bootstrap reduces when more datasets are used (left to right denotes the increase in number of datasets for prediction). The confidence interval also reduces with the increase in the number of datasets used. For instance, the 0.632 bootstrap error using gene expression data is 0.163 for Drug 1 with 80% confidence interval of 0 to 0.87 whereas use of all five datasets reduces the 0.632 bootsrap error to 0.075 with 80% confidence interval of 0 to 0.26. Drug 10 also presents similar results where the 0.632Bootstrap error reduces from 0.21 for gene expression to 0.066 for all datasets combined. The confidence interval is also compressed more than 3.5 times by using all the datasets as compared to gene expression alone. We also applied our framework for predicting the sensitivity of the 18 test cell lines for drug 1 whose gold standard results were provided following the end of the challenge. When we used gene expression dataset alone for training and prediction, the root mean square (RMS) error is 0.2087 whereas using all the five datasets produces an RMS error of 0.1628. The results signify the ability of our framework to lower the mean absolute error along with the associated variance of the error estimation by incorporating multiple datasets. To explore the importance of linear regression part in our framework for combining the predictions from different datasets, we considered the simple mean of predictions from each dataset for drug 1 and obtained a 0.632 bootstrap error of 0.234 for combining 5 datasets which is much higher than 0.075 obtained through our linear regression framework.

**Figure 7:**
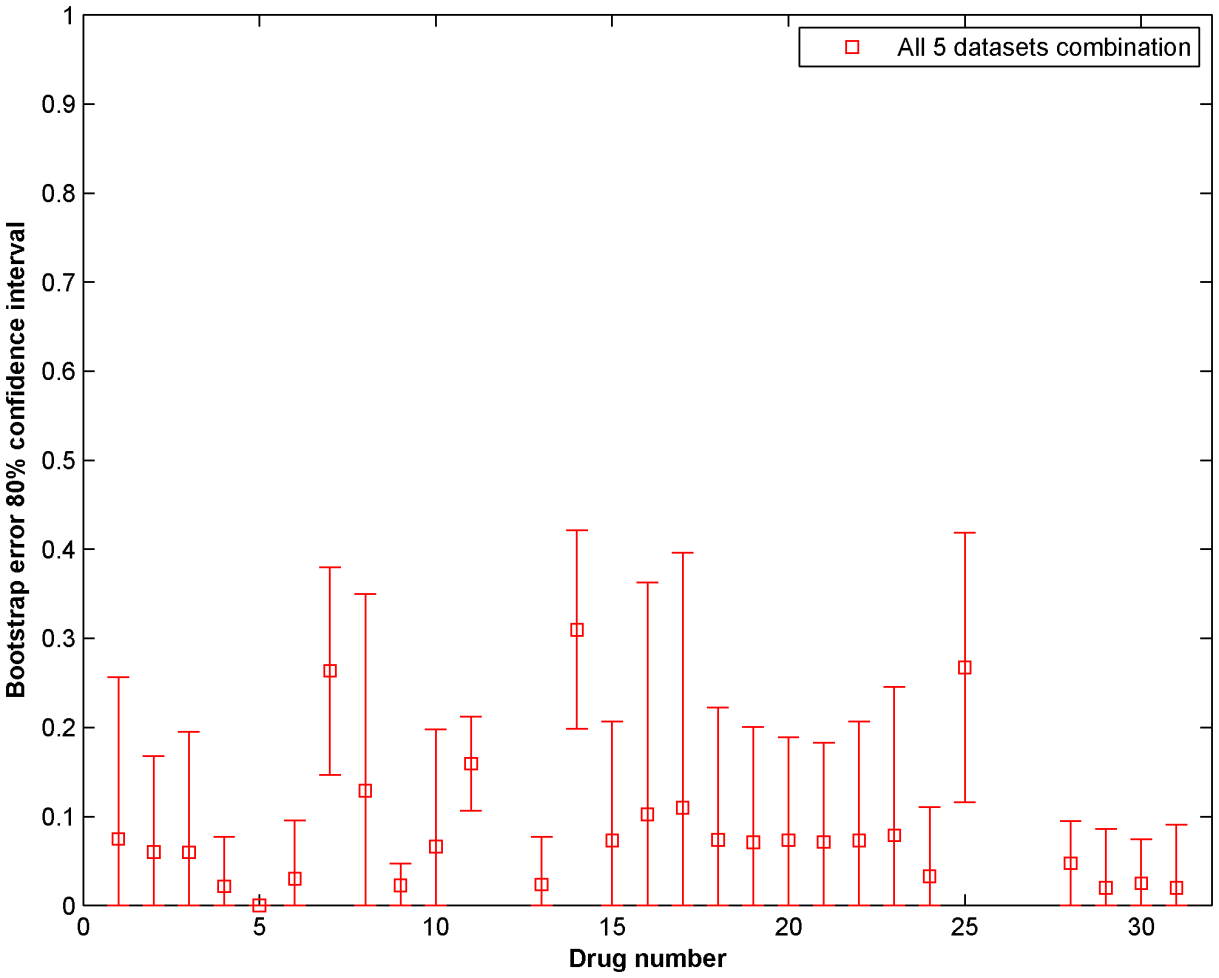
Mean 0.632 Bootstrap error and 80% confidence intervals for Drugs 1 to 31 considering all 5 datasets for prediction. The prediction for drugs 12, 26 and 27 were not considered as they contained minimal variations in sensitivity.

**Figure 8:**
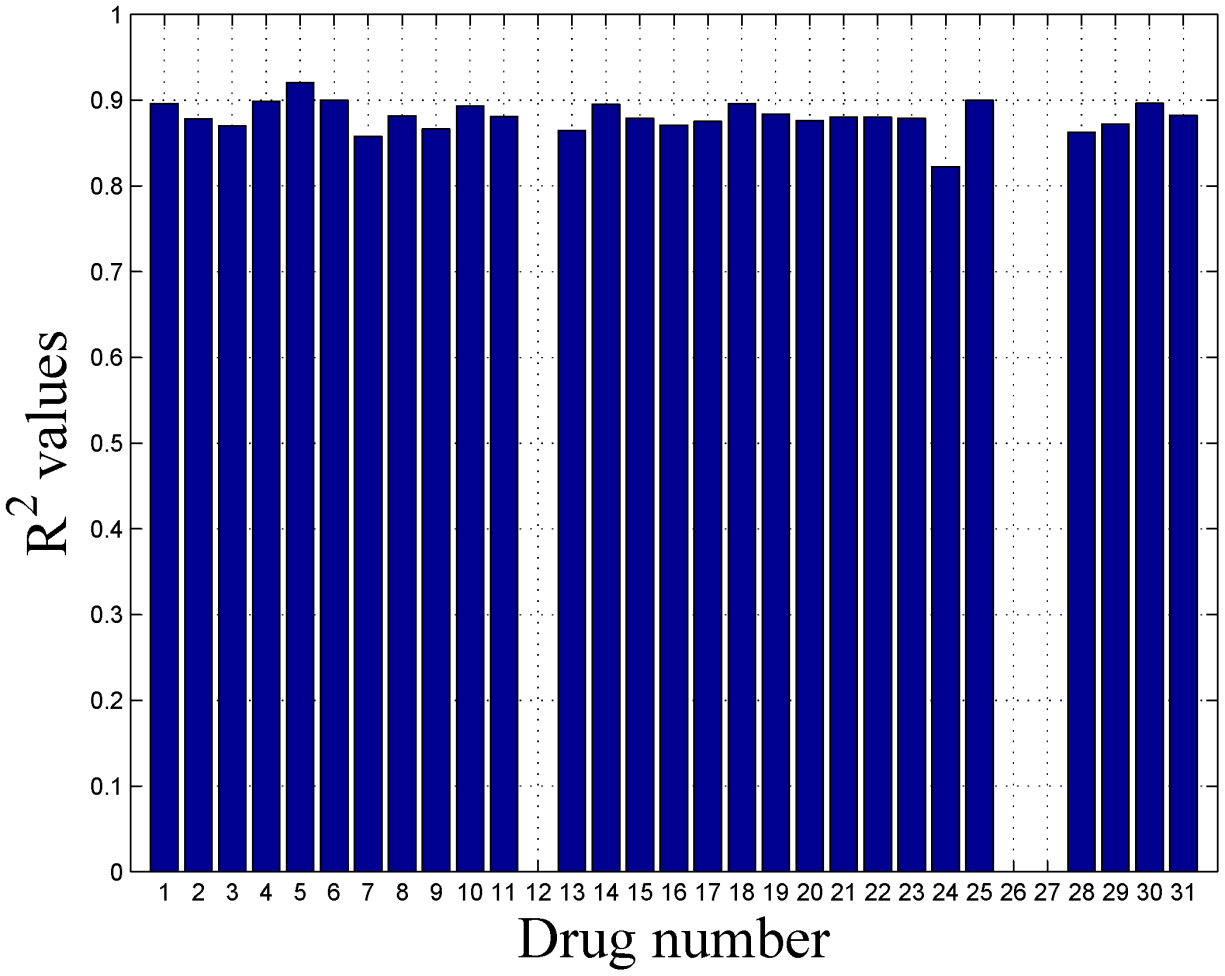
The coefficient of determination 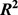 between predicted and experimental sensitivities using bootstrap samples for Drugs 1 to 31 while considering all 5 datasets for prediction. The prediction for drugs 12, 26 and 27 were not considered as they contained minimal variations in sensitivity.

Figure 7 shows the 0.632 Bootstrap error and 80% confidence intervals for Drugs 1 to 31 considering all 5 datasets for prediction. The prediction for drugs 12, 26 and 27 were not considered as they contained minimal variations in sensitivity. The regression coefficients of the five individual genomic characterization datasets in the integrated modeling framework are included as information S2. Figure 8 shows the coefficient of determination 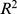 between predicted and experimental sensitivities using bootstrap samples for drugs 1 to 31 while considering all 5 datasets for prediction. We should note that the 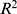 values are above 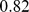 for all the drugs denoting good prediction accuracy while using five genomic characterization datasets.

### Analysis of CCLE Datasets

In this sub-section we present results for applying our framework to CCLE [18] datasets. We used two types of genomic characterization (Gene Expression and SNP6) for our integrated prediction. We considered 10-fold cross validation for error estimation as the number of cell lines was relatively large (300 to 500 for each drug). For this analysis, we also considered the top predictors based on the integrated random forest model. The top predictors for individual Random Forests were generated based on the average bootstrap error of the trees in the forest containing the specific feature. The combined model regression coefficients were used to generate the top predictors of the joint model from the predictor weights of the individual models. Some of the top predictors were then validated by comparing with the top targets of the drugs that can be obtained from drug target inhibition profiles [19], [21]. Note that not all top predictors will be targets of a drug as the expression of upstream or downstream proteins of the drug targets may predict the efficacy of a drug without being actual targets of the drug.

For the current analysis, the Gene Expression and SNP6 datasets consists of 18,988 and 21,217 genes respectively and drug responses for around 500 cell lines is available for the following 24 drugs *17AAG, AEW541, AZD0530 (Saracatinib), AZD6244 (Selumetinib), Erlotinib, Irinotecan,L685458, Lapatinib, LBW242, Nilotinib, Nutlin3, Paclitaxel, Panobinostat, PD0325901, PD0332991, PF2341066 (Crizotinib), PHA665752, PLX4720, RAF265, Sorafenib, TAE684, TKI258 (dovitinib), Topotecan and ZD6474 (Vandetanib)*. Since majority of the applied drugs target the human kinome (the set of protein kinases), we also consider a smaller set of features containing around 400 kinase producing genes. The 400 kinases are selected from drug target inhibition profile studies [19], [20]. We report our prediction results in the form of correlation coefficients between predicted and experimental sensitivities to directly compare our results with the recently published CCLE study [7]. We have also used 10 fold cross validation similar to [7]. The CCLE study however used multiple other data types (for a total of 150,000 features) but we have used only gene expression and SNP6. The results of our prediction approach are shown in Table 4. We note that the average correlation coefficient across the 24 drugs was 0.421 for the CCLE Elastic net study [7] but our approach based on only 400 features produced a higher average correlation coefficient of 0.454 (we will term this approach *CRF-400*), an increase of 7.8%. When we used all the features (18,988 gene expression features and 21,217 SNP6 features), we were able to increase our average correlation coefficient to 0.473 (we will term this approach *CRF-20,000*), an increase of 12.4%. We next applied our framework to select the top predictors for each drug for both CRF-400 and CRF-20,000. The details on the top predictors are available as information S3. The top 20 predictors are then compared with the experimentally validated targets of the drugs based on earlier studies [19], [21]. For instance, the top predictor for the drug Erlotinib based on both CRF-400 and CRF-20000 is EGFR and EGFR is known to be the primary target of Erlotinib with an 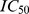 of 0.2 nM [19]. 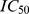 denotes the drug concentration required to inhibit the target protein expression by half. If we consider the drug Lapatinib, the top predictor selected was ERBB2 for both *CRF-400* and *CRF-20000*. ERBB2 is the most potent target of Lapatinib (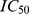 of 9.2nM [20]) followed by EGFR (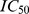 of 10.8 nM [20]). However, EGFR was selected as the 34th top predictor for *CRF-400* and was not picked up by *CRF-20,000*. The analysis of the top predictors shows that *CRF-400* has better chances of selecting the experimentally validated primary targets of a drug in the top 20 predictors as compared to *CRF-20,000*. However, the prediction accuracy is decreased on an average by 4% by using the smaller set of 400 features. Our ability to select the top drug targets using *CRF-20,000* might be increased if we use more number of trees so that each feature appears in a large number of trees in the forest. Furthermore, the inclusion of primary drug targets in the top predictors of the model will also depend on the dataset. For instance, if we consider the individual Random Forest (400 features) models for Gene Expression and SNP6 for Erlotinib, EGFR is selected as the top predictor for Gene expression but PHKG1 is selected as the top predictor for SNP6. But since the regression weights in the combined models are 0.89 for gene expression and 0.11 for SNP6, the top predictor for the combination model is EGFR. The analysis of the CCLE database illustrates the predictive capability of the integrated random forest approach along with the ability to generate top predictors for drug sensitivity.

**Table 4:** CCLE Drug sensitivity prediction results in the form of correlation coefficients between experimental and predicted sensitivities.

	Correlation Co-efficients		
Drug Name	Elastic Net [7]	CRF-400	CRF-20,000
17AAG	0.43	0.4116	0.4397
AEW541	0.33	0.4037	0.3934
AZD0530 (Saracatinib)	0.18	0.2855	0.2747
AZD6244 (Selumetinib)	0.58	0.516	0.5909
Erlotinib	0.3	0.4034	0.4333
Irinotecan	0.68	0.6214	0.6776
L685458	0.47	0.5351	0.5423
Lapatinib	0.45	0.5488	0.5263
LBW242	0.08	0.184	0.1400
Nilotinib	0.76	0.5458	0.5476
Nutlin3	0.1	0.2892	0.3096
Paclitaxel	0.6	0.5453	0.5531
Panobinostat	0.65	0.616	0.6503
PD0325901	0.64	0.5837	0.6471
PD0332991	0.58	0.5077	0.5141
PF2341066 (Crizotinib)	0.36	0.5121	0.5055
PHA665752	0.27	0.3393	0.3437
PLX4720	0.55	0.4459	0.4768
RAF265	0.35	0.4378	0.4394
Sorafenib	0.27	0.4099	0.4685
TAE684	0.35	0.4073	0.4453
TKI258 (dovitinib)	0.3	0.4463	0.4611
Topotecan	0.58	0.5619	0.6226
ZD6474 (Vandetanib)	0.24	0.3331	0.3494
Average	0.421	0.454	0.473

Elastic Net denotes the approach applied in [7] for predicting sensitivity using 10-fold cross validation from CCLE database. CRF-400 denotes our proposed combined Random Forest approach using gene expression and SNP6 data of only 400 genes. CRF-20000 denotes our proposed combined Random Forest approach using 18,988 gene expression and 21,217 SNP6 features.

## Discussion

In this article, we presented an ensemble based approach for predicting drug sensitivity from genomic characterizations. Predictions from individual datasets were combined in a linearly weighted fashion to generate the integrated prediction. The combined prediction could have been explored in multiple other ways such as integrating the datasets before designing a learner. However, the current approach was able to produce high accuracy prediction for both the initial NCI-DREAM drug sensitivity challenge and the second round of evaluation. The final weighted averaging of predictions from different datasets can be considered as a continuation of ensemble approach where the results from different forests are combined. Our results show that the Random Forest approach with averaging over multiple regression trees can produce robust predictions from individual datasets. Based on this general idea, we expected the weighted combination of the predictions from different forests to produce robust prediction results. Previous DREAM challenges on inference of genetic regulatory networks have shown that combining predictions from multiple inference approaches can produce robust prediction results [22] and the presented approach to drug sensitivity prediction also combines predictions from multiple learners trained on multiple data types.

Biological data collection often suffers from noise in measurements and missing information for some of the samples. Thus combining predictions from multiple learners with low correlation between themselves is expected to reduce the effect of noise and avoid over-fitting. For deciding on the joint model or one of the individual models for each drug, we relied on leave-one-out error estimation to guide the selection process. Even though leave-one-out error estimation can have high variance as compared to the true error, time and sample limitations directed our choice for the challenge submission. In this article, we also presented the error estimation using 0.632 Bootstrap and error confidence intervals using jackknife-after-bootstrap. For 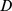 different genomic or epigenetic characterizations, we can generate 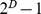 different non-null combination predictions. A form of error estimation such as leave-one-out or 0.632 Bootstrap can then be applied to decide the best combination rather than selecting the prediction from all the 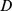 datasets. Some of the datasets such as mutation data may not be informative enough for predicting the drug sensitivity alone, they can then possibly be combined with other data sets to aid in prediction. For instance, the probability of selecting the expression from a mutated gene in prediction can be increased to provide higher weights to genes with existing mutations. Our results indicate that the current framework is suitable in reducing the 0.632 bootstrap error for combining multiple datasets as shown in figures 5 and 6 where mean 0.632 bootstrap error and error variance reduces with increase in different genomic characterizations for prediction.

For the NCI-DREAM challenge results, we had applied the prediction approach for each drug separately. For the second round of evaluation, we had to predict the response to 12 drugs based on the genomic characterizations of the cell lines and we could have used the response of the cell lines to the earlier 31 drugs (the gold standard for the first round of challenge). The inclusion of the response to the earlier drugs as 31 additional features in a Random Forest didn’t noticeably improve our accuracy (as estimated by leave-one-out errors) and thus our predictions for second round of evaluation were based on genomic characterizations alone. However, if more information on the drugs are provided such as the 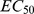 s of individual targets of the drugs, the response of a cell line to drugs with known target inhibition profiles can be extremely useful in predicting the response to a new drug with known target inhibition profile [23]. The target inhibition profile refers to the percent inhibition of different targets (such as Kinases) at the applied drug concentration [19], [21].

## Materials and Methods

**Figure 9:**
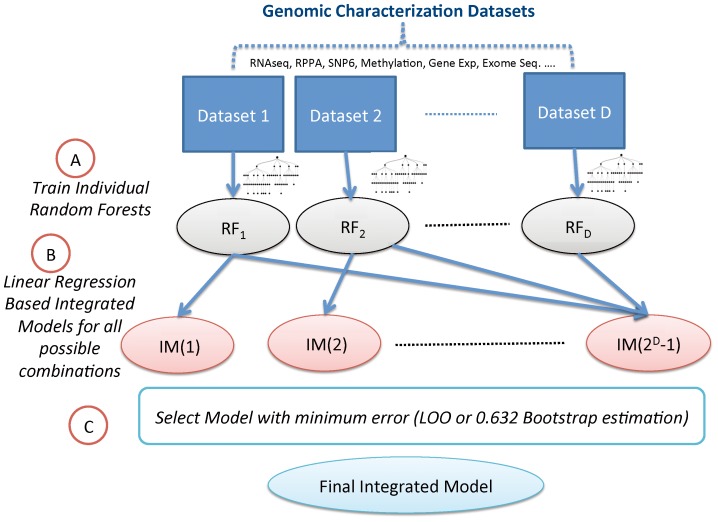
Integrated Model Design Workflow: (A) Random Forest models based on individual genomic characterizations are generated (B) Integrated models are generated based on different combinations of individual models using least squares regression for estimating the model weights (C) the combination with lowest error as estimated using leave one out (LOO) or 0.632 Bootstrap error estimation is selected.

Let us consider that we have 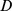 types of heterogeneous genomic characterizations denoted by 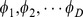. For pre-processing of the genomic data, any missing value was estimated by averaging the two nearest data points. Other missing value estimation approaches can also be applied [24]. Let 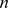 denote the number of samples available for training the predictive models with given drug response 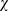. Our approach to generate the final combined models consists of the following (a) Generate 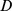 Random Forest based predictive models from each genomic characterization dataset (the details are provided in the next sub-section) (b) Design linearly weighted integrated models from the 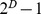 possible combination of datasets using least square regression for estimating the model weights. (c) Estimate the efficacy of the combination models based on 0.632 Bootstrap or Leave-one-out error estimation. The combination model with the smallest error is selected as the final integrated model for future predictions. The steps in the design of the integrated model are shown graphically in figure 9.

### Random Forest Approach

Random Forest regression refers to ensembles of regression trees [25]. A set of 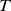 un-pruned regression trees are generated based on bootstrap sampling from the original training data. We considered 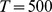. For each node, a random set of 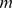 features selected from the total features (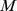) is used for fitting a regression tree based on the bootstrap sample. We considered 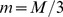. Since the number of important features for predicting the drug sensitivity maybe a small fraction of the overall set of features, we considered a large 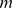 to avoid missing the important features during the randomized feature selection process. For instance, if the number of important features is denoted 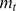, the probability 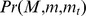 of not selecting any of the important features in one randomized variable selection process is given by 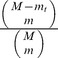. As a numerical example, with 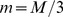, we have 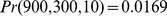 and a smaller 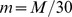 results in a higher probability 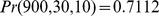 of not selecting any of the important features in any random node. However, higher 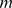 can increase the correlation between the regression trees and thus possibly increase the variance of the overall random forest prediction.

Using the randomized feature selection process, we fit the tree based on the bootstrap sample 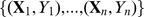 generated from the training data. During the tree generation process, a node with less than 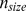 training samples is not partitioned any further. We selected 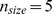. Let us consider the prediction based on a test sample 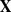 for the tree 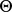. Let 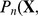 θ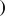 be the partition containing **X**, the tree response takes the form [25], [27]

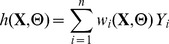
(1)where the weights 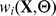 are given by



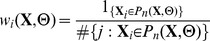
(2)Let the 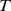 trees of the Random forest be denoted by 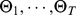 and let 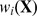 denote the average weights over the forest i.e.
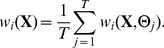
(3)


The Random Forest prediction for the test sample 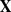 is then given by
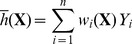
(4)


### Integration of predictions from multiple data-types

Let 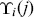 denote the prediction obtained by Random Forest approach for genomic characterization dataset 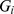 and cell line 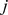. To utilize the biological information in different datasets for prediction, we consider a linearly weighted combination model. We use least square regression to estimate the weights for each dataset 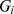 by minimizing
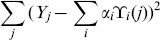
where 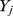 is the experimental drug response for cell line *j*, 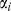 is the corresponding weight of dataset 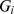.

Following the generation of the weights of the individual datasets, the combined prediction result 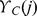 is generated as follows:
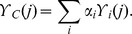



### Normalization and error calculation

Following the generation of predictions from our integrated random forest approach, we normalized the predicted and experimental drug sensitivities between 0.0 and 1.0 by min-max normalization [28]. The normalized prediction 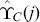 and actual drug response 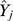 were calculated as follows:
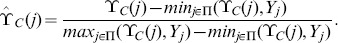





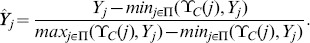



where 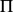 denotes the set of all available cell lines. After normalization, the corresponding error 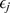 of cell line *j* was generated as 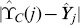, both for the bootstrap error 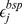 and the leave-one-out error 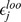.

Consider 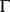 bootstrap samples created from the 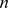 samples 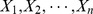. Let 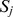 the set of bootstrap samples not containing cell line *j*, then the overall bootstrap error of cell line *j* can be computed as follows:
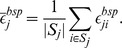



The corresponding 0.632 bootstrap error can be computed as 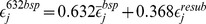, where 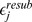 denotes the re-substitution error for cell line *j*. For re-substitution error calculations, all the cell lines were used for training and testing the integrated Random Forest model.

For leave-one-out error calculations, let 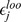 denote the normalized error in prediction when the set of cell lines 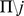 were used for training the integrated Random Forest model and tested on the cell line 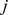. The average leave-one-out error across all cell lines for a drug is calculated as follows:
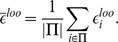



### Generation of Confidence Intervals

For detailed analysis of DREAM challenge datasets, we employed Jackknife-After-Bootstrap approach [16], [17] for generating the confidence intervals of the 0.632 Bootstrap errors. Let 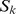 denote the set of bootstrap samples that do not contain sample 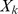 and denote by 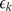, the 0.632 bootstrap estimate computed from 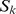. The standard error can be computed as
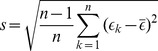
(5)where 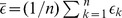. The 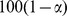 % prediction intervals for the true error can be computed as [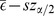, 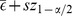] where 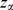 is the 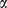 quantile of the standard normal distribution. Since we consider the absolute error, the lower bound of the confidence interval is calculated as 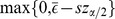.

### DREAM challenge evaluation approach

The predictions of our model, as well as of other participants in NCI-DREAM drug sensitivity prediction challenge were evaluated in terms of an overall score (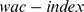 [13]) based on actual experimentally determined cell-line rankings. Since the actual ranking for a given drug is subject to uncertainties, a pooled standard deviation 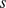 for each drug is used to account for these uncertainties. The pooled standard deviations were measured drug-wise and independent of the cell lines. For each drug, an individual score (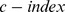) of the concordance between the experimental and predicted ranking lists was calculated using the Gauss error function 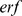. Specifically, let 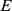 and 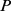 denote the experimental and predicted ranking vectors, respectively. For cell line pair 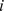 and 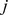, a score 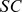 is computed as follows: 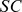 = 0.5(1+erf[(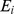 − 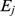)/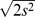]). For the case of 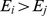 and 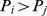, 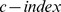 is increased by 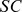; otherwise if 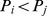, it is increased by 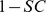. Furthermore, if 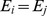 while 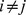, 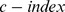 is added a score of 0.5. The final 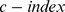 for each drug is normalized and a larger score indicates greater statistical significance of the prediction.

After obtaining the 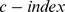 for individual drugs, an overall score was calculated as the weighted average across all the 31 drugs. To compute the weight 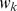 for drug 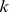, an empirical null distribution of 10,000 random sets of predictions was generated. Let 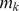 and 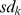 denote the mean and standard deviation of the random predictions, 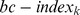 indicates the best 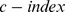 acquired from the experimentally determined ranking, then 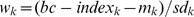. Eventually, a team’s overall score 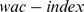 can be defined as 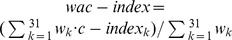.

## Supporting Information

Information S1 **Drug Sensitivity Rankings.**
(XLSX)

Information S2 **Regression coefficients of individual genomic characterization datasets in the final integrated model for DREAM Challenge provided datasets.**
(XLSX)

Information S3 **Top predictors based on gene expression and SNP6 integrated random forest model for CCLE data.** Two cases are considered: CRF-400 where only 400 genes from the kinome are used in the predictive model (i.e. 400 features for each SNP6 and gene expression dataset) and CRF-20,000 where all the 18,988 gene expression and 21,217 SNP6 features are utilized.
(XLSX)
